# Perceived Stress and Subjective Auditory Sensitivity in Autistic and Non-Autistic University Students: A Cross-Sectional Comparative Study

**DOI:** 10.3390/healthcare14193130

**Published:** 2026-09-22

**Authors:** Miguel Fuentes-Contreras, Francisca Galindo-Veroiza, Cristian Núñez-Espinosa, Nicolás Gómez-Ampuero, Carlos Alcayaga-Palazón, Bárbara Correa-Toledo

**Affiliations:** 1 Speech, Language, and Hearing Sciences Program, Universidad de Magallanes, Punta Arenas P.O. Box 113-D, Chile; nicolas.gomez@umag.cl (N.G.-A.); bcorrea@umag.cl (B.C.-T.); 2 Department of Psychology, Universidad de Magallanes, Punta Arenas P.O. Box 113-D, Chile; veroizagalindo@gmail.com; 3 School of Medicine, Universidad de Magallanes, Punta Arenas P.O. Box 113-D, Chile; cristian.nunez@umag.cl (C.N.-E.); carlos.alcayaga@umag.cl (C.A.-P.)

**Keywords:** autism spectrum disorder, autistic students, auditory sensitivity, hyperacusis, perceived stress, sensory processing, higher education

## Abstract

**Highlights:**

**What are the main findings?**

- University students with autism spectrum disorder (ASD) reported significantly
- higher levels of perceived stress and subjective auditory sensitivity than non-autistic students.
- Positive associations between perceived stress and subjective auditory sensitivity were observed in both groups, although students with ASD showed substantially greater auditory vulnerability.

**What are the implications of the main findings?**

- Perceived stress was positively associated with subjective auditory sensitivity but did not fully explain the elevated auditory sensitivity observed in university students with ASD.
- Auditory sensitivity should be considered a relevant component of well-being and academic adaptation in autistic university students and may help inform the development of sensory-aware support strategies in higher education settings.

**Abstract:**

Autistic individuals frequently report auditory hypersensitivity, which may affect academic participation, social functioning, and well-being. However, the association between perceived stress and subjective auditory sensitivity remains insufficiently explored in autistic university students, a population exposed to high cognitive, social, and sensory demands. **Objective:** This study examined the association between perceived stress and subjective auditory sensitivity in autistic and non-autistic university students. **Methods:** This cross-sectional comparative study included 68 university students: 30 with a formally documented ASD diagnosis and 38 without self-reported ASD, neurological, or auditory disorders. Participants completed the Perceived Stress Scale-14 and the Khalfa Auditory Sensitivity Questionnaire. Between-group comparisons were performed using Mann–Whitney U tests, and associations were examined using Spearman’s rho correlations. **Results:** The ASD group showed significantly higher scores in perceived stress and total subjective auditory sensitivity than the comparative group (*p* < 0.001). Likewise, significantly higher scores were observed in all Khalfa questionnaire subscales among participants with ASD (*p* < 0.001). In the ASD group, moderate positive correlations were identified between perceived stress and total subjective auditory sensitivity (rho = 0.39; *p* = 0.035), as well as the attentional dimension (rho = 0.39; *p* = 0.034). In the comparative group, significant positive correlations were observed between perceived stress and total subjective auditory sensitivity (rho = 0.62; *p* < 0.001), as well as all Khalfa subscales. Although auditory sensitivity scores were markedly higher in the ASD group, the association between perceived stress and auditory sensitivity was stronger and more consistent across subscales in the comparative group. **Conclusions:** Perceived stress is associated with subjective auditory sensitivity in university students, regardless of diagnostic status. However, the markedly higher auditory sensitivity observed among autistic students may reflect a broader sensory vulnerability not fully explained by perceived stress alone.

## 1. Introduction

Autism spectrum disorder (ASD) is a neurodevelopmental condition characterized by difficulties in communication and social interaction, together with restricted or repetitive behavioral patterns and interests. However, in addition to these core characteristics, a substantial proportion of people with ASD present alterations in sensory processing, with increased or decreased responses to environmental stimuli, particularly in the auditory domain [1]. These sensory alterations may translate into experiences of environmental overload and difficulties in adequately modulating environmental information, significantly affecting everyday well-being, social adaptation, and academic performance. In this context, increased sensitivity to auditory stimuli is one of the most frequently reported sensory manifestations in people with ASD. It may generate discomfort, avoidance behaviors, and greater vulnerability to environments perceived as highly demanding from a sensory perspective [2].

In higher education contexts, these sensory features may become particularly relevant because university students are frequently exposed to crowded classrooms, unpredictable acoustic environments, social interaction demands, and sustained cognitive load. For autistic students, these conditions may increase perceived environmental burden and interfere with academic participation, concentration, and emotional well-being.

Among the sensory alterations described in people with ASD, increased auditory sensitivity is one of the most frequent and clinically relevant manifestations. This condition is characterized by an exacerbated perception or low tolerance to everyday sounds that, for most people, do not generate significant discomfort [3]. The presence of subjective auditory sensitivity may be associated with discomfort responses, avoidance of certain environments, and difficulties in social and academic interaction, significantly affecting quality of life and functional performance in those who experience it [4]. Likewise, studies in populations with ASD have described that auditory alterations are related not only to the intensity of the sound stimulus, but also to difficulties in the modulation, filtering, and processing of auditory information, favoring states of sensory overload and increased vigilance toward the acoustic environment [2].

Auditory perception and tolerance to sound stimuli depend on complex mechanisms of neural processing and modulation that involve both central and peripheral structures of the auditory system. At the central level, regions such as the thalamus, auditory cortex, and limbic system structures actively participate in the integration, interpretation, and emotional response to sound, influencing the subjective perception of auditory discomfort or threat [5]. In parallel, the efferent auditory system, particularly the medial olivocochlear pathway, plays a fundamental role in regulating auditory sensitivity through mechanisms of gain control and inhibition of cochlear amplification, thereby modulating responses to environmental stimuli and facilitating auditory discrimination in complex contexts. Direct recordings in awake animals have recently confirmed that this efferent pathway actively adjusts cochlear amplification and may compensate for reduced auditory sensitivity, a mechanism that has been proposed as clinically relevant to conditions such as hyperacusis and tinnitus. Alterations in these modulation mechanisms could favor states of auditory hyperreactivity and difficulties adapting to sound environments, especially in individuals with greater sensory vulnerability [6].

Several studies have indicated that stress can significantly influence the perception and processing of sensory stimuli, favoring states of hyperreactivity to the environment. At the neurophysiological level, stress responses involve the activation of autonomic and neuroendocrine systems that can modify the activity of structures involved in auditory perception and the emotional regulation of sound, including limbic regions and mechanisms of descending auditory modulation [7]. In this context, sustained increases in vigilance and physiological activation could enhance sensitivity to everyday auditory stimuli, thereby increasing the perception of sound-related discomfort. In people with ASD, this interaction could be especially relevant due to pre-existing alterations in sensory processing and difficulties regulating environmental stimuli, favoring states of auditory overload and pre-existing stress [2].

Stress-related neurophysiological models suggest that sustained physiological arousal may modulate auditory processing and increase the perceived salience or discomfort of sounds. Mechanisms involving limbic activation, descending auditory modulation, and peripheral auditory neurotransmission have been proposed, although their relevance in autistic adults remains insufficiently tested. Therefore, these models provide a plausible but still exploratory framework for examining the stress–auditory sensitivity relationship. Sahley and Nodar proposed a neurochemical model to explain how stress could promote increased auditory sensitivity via peripheral mechanisms of neural modulation [8].

In people with ASD, the interaction between stress and auditory sensitivity may be particularly relevant given the coexistence of alterations in sensory processing, difficulties in modulating environmental stimuli, and greater susceptibility to perceptual overload. Several studies have described that people with ASD show an increased tendency toward hypervigilance and amplified responses to everyday auditory stimuli, which could favor persistent experiences of sound discomfort, cognitive fatigue, and impairment in social interaction. From this perspective, neurophysiological mechanisms associated with stress could further potentiate the subjective perception of auditory sensitivity in this population, particularly given prior functional differences in systems of emotional regulation, attention, and sensory processing. Consequently, subjective auditory sensitivity in people with ASD could be understood as a multidimensional phenomenon influenced by both peripheral and central auditory mechanisms and by emotional and physiological processes associated with stress [2,9].

Despite the available evidence, three relevant limitations remain. First, much of the research on auditory sensitivity in ASD has focused on child or clinical populations, with less attention to university students, who face academic environments characterized by high cognitive load, social exposure, and constant sensory demands. Second, although stress has been proposed as a modulator of sensory experience, few studies have directly evaluated its association with subjective auditory sensitivity in young adults with ASD. Third, most studies do not include a comparative group without ASD, making it difficult to establish whether the relationship between stress and auditory sensitivity represents a general pattern or a differential association in autistic populations.

To address these gaps, the present study examined whether perceived stress is associated with subjective auditory sensitivity in autistic and non-autistic university students. We hypothesized that autistic students would report higher perceived stress and subjective auditory sensitivity than non-autistic students, and that perceived stress would be positively associated with subjective auditory sensitivity in both groups. Because auditory sensitivity in autism may reflect a more stable sensory processing vulnerability, we further explored whether the strength of this association differed between groups.

## 2. Materials and Methods

### 2.1. Study Design

An observational, cross-sectional, comparative, and correlational study was conducted to analyze the relationship between perceived stress and subjective auditory sensitivity in university students diagnosed with autism spectrum disorder (ASD), also incorporating a comparative group without an ASD diagnosis. The study was conducted among students at the Universidad de Magallanes in Punta Arenas, Chile.

### 2.2. Participants

The sample was obtained through non-probabilistic convenience sampling and comprised 68 university students aged 18 to 40 years. The ASD group comprised 30 students with a formal diagnosis of autism spectrum disorder, while the comparative group comprised 38 students without an ASD diagnosis. The absence of an ASD diagnosis was determined through participant self-report during the eligibility screening questionnaire, and no participant reported a previous clinical diagnosis of autism spectrum disorder. The comparative group was recruited from the same university context but was not individually matched to the autistic group by sex, academic program, or mental health status. Therefore, between-group comparisons should be interpreted considering the possibility of residual confounding.

For inclusion in the ASD group, participants were required to have a formal diagnosis of autism spectrum disorder and to be regular students at the Universidad de Magallanes. Diagnostic verification was performed using records previously accredited by the university’s Pedagogical Support Unit for People with Disabilities. For the comparative group, university students without an ASD diagnosis and with no self-reported history of neurological or auditory disorders were included. Participants who reported a previous diagnosis of hearing loss, active auditory pathology, current use of hearing devices, major neurological history, or medical conditions that could directly interfere with auditory perception or responses to the questionnaires were excluded. Given the exploratory nature of the study, participants were not excluded based on anxiety or other mental health conditions, although this information should be considered a possible source of uncontrolled confounding.

Given the exploratory nature of the study and the limited availability of formally diagnosed autistic university students, no a priori sample size calculation was performed. Therefore, the findings should be interpreted as preliminary and hypothesis-generating.

### 2.3. Instruments

#### 2.3.1. Perceived Stress Scale (PSS-14)

To assess perceived stress levels, the Perceived Stress Scale (PSS-14), developed by Cohen, Kamarck, and Mermelstein (1983) [10] and adapted and validated in the Chilean population [11], was used. This instrument measures the extent to which people perceive daily life situations as unpredictable, uncontrollable, or overwhelming. The scale consists of 14 items with a five-point Likert response format, scored from 0 (“never”) to 4 (“very often”). A higher total score indicates a higher level of perceived stress. The Chilean-validated version has demonstrated adequate psychometric properties in previous studies. In the present sample, internal consistency was estimated using Cronbach’s alpha.

#### 2.3.2. Khalfa Auditory Sensitivity Questionnaire

Subjective auditory sensitivity was assessed using the questionnaire proposed by Khalfa, Veuillet, Pérez-Díaz, Jouvent, and Collet (2002) [4], an instrument designed to explore subjective reactions to everyday auditory stimuli and environmental sounds. The original questionnaire was developed and validated in a sample of 201 adults randomly recruited from the general population, predominantly university students, without specific selection criteria regarding auditory status or clinical condition. In the present study, the validated Spanish version of the questionnaire was used. Representative items assess difficulties concentrating or reading in noisy environments, tolerance and understanding of speech in noise, the social impact of noise exposure, and emotional reactions to everyday sounds.

The questionnaire consists of 14 items with a four-point Likert response scale, scored from 0 (“no”) to 3 (“yes”). Higher scores reflect greater perceived subjective auditory sensitivity. For the dimensional analysis, the instrument’s proposed subscales were used: attentional subscale (items 1–4), social subscale (items 5–10), and emotional subscale (items 11–14).

### 2.4. Procedure

Participants were recruited through institutional online dissemination and contact with students registered in the Pedagogical Support Unit for People with Disabilities at the Universidad de Magallanes.

Subsequently, participants completed an online form that included the electronic informed consent, general background information, the PSS-14 and the Khalfa Auditory Sensitivity Questionnaire.

### 2.5. Statistical Analysis

Statistical analysis was performed using IBM SPSS Statistics software, version 24.

Initially, descriptive statistics were calculated for all study variables, including means, standard deviations, minimum and maximum values, skewness, and kurtosis. The normality of the distributions was subsequently assessed using the Shapiro–Wilk test to determine the most appropriate inferential statistical approach. Because several of the analyzed variables did not meet the assumption of normality, non-parametric tests were used for the inferential analyses.

Between-group comparisons were performed using the Mann-Whitney U test because several variables did not meet the normality assumption. To complement significance testing, the effect size was estimated for between-group comparisons. The relationship between perceived stress and subjective auditory sensitivity was examined using Spearman’s rho, reporting the magnitude, direction, and significance of the association. The internal consistency of the instruments was evaluated using Cronbach’s alpha coefficient for the PSS-14 scale, the total score of the Khalfa questionnaire, and its subscales. This analysis was performed to estimate the reliability of the measurements in the studied sample.

Correlation coefficients were interpreted according to conventional thresholds as weak, moderate, or strong associations. A statistical significance level of *p* < 0.05 was considered.

### 2.6. Ethical Considerations

The study was approved by the Scientific Ethics Committee of the Universidad de Magallanes. All participants voluntarily provided electronic informed consent before participation. Participation was voluntary and anonymous, and the confidentiality of all collected information was safeguarded throughout the research process.

## 3. Results

### 3.1. General Characteristics of the Sample

The sample consisted of 68 university students, distributed into a group diagnosed with autism spectrum disorder (ASD) (*n* = 30) and a comparative group without an ASD diagnosis (*n* = 38). Participants in the ASD group had a mean age of 22.8 ± 5.0 years (median [IQR]: 22 [19–25]), whereas participants in the comparative group had a mean age of 22.1 ± 4.9 years (median [IQR]: 21 [20–22]). In the ASD group, 56.7% of participants were female, whereas the comparative group showed a balanced distribution between female and male participants.

No significant differences were observed between the ASD and comparative groups regarding age (Mann–Whitney U = 521.50, Z = −0.61, *p* = 0.545) or sex distribution (Fisher–Freeman–Halton exact test, *p* = 0.460). Age was not significantly correlated with perceived stress (Spearman’s rho = −0.13, *p* = 0.282) or subjective auditory sensitivity (Spearman’s rho = 0.12, *p* = 0.319), suggesting that it was unlikely to represent a relevant confounding factor in the present sample.

Assessment of the distributional characteristics indicated that several variables did not satisfy the assumption of normality, supporting the use of non-parametric statistical analyses.

The internal consistency of the study instruments was evaluated using Cronbach’s alpha coefficients. The PSS-14 demonstrated good internal consistency (α = 0.87), whereas the Khalfa Auditory Sensitivity Questionnaire demonstrated excellent internal consistency (α = 0.96). Cronbach’s alpha values for the Khalfa attentional, social, and emotional subscales were 0.83, 0.93, and 0.90, respectively. The ASD group showed significantly higher perceived stress scores than the comparative group (U = 249.50, Z = −3.97, *p* < 0.001, r = 0.48). Likewise, the ASD group showed significantly higher scores in total subjective auditory sensitivity and in all subscales of the Khalfa questionnaire compared with the comparative group (all *p* < 0.001), with moderate-to-large effect sizes observed across comparisons (Table 1).

### 3.2. Correlation Between Perceived Stress and Subjective Auditory Sensitivity

In the ASD group, moderate positive correlations were observed between perceived stress scores (PSS-14) and the attentional dimension (rho = 0.39; *p* = 0.034), as well as with the total score of the Khalfa questionnaire (rho = 0.39; *p* = 0.035). Positive correlations were also observed for the social (rho = 0.31; *p* = 0.098) and emotional (rho = 0.28; *p* = 0.129) dimensions; however, these associations did not reach statistical significance.

In the comparative group, significant positive correlations were observed between perceived stress and total subjective auditory sensitivity (rho = 0.62; *p* < 0.001), as well as with the attentional (rho = 0.43; *p* = 0.007), social (rho = 0.52; *p* = 0.001), and emotional (rho = 0.61; *p* < 0.001) dimensions (Table 2).

The correlation coefficient between perceived stress and total subjective auditory sensitivity was numerically higher in the comparative group (rho = 0.62) than in the ASD group (rho = 0.39). However, no formal statistical comparison between correlation coefficients was performed; therefore, this numerical difference should be interpreted descriptively. These findings should be interpreted descriptively rather than as evidence of statistically different correlation strengths between groups. In contrast, in autistic students, elevated auditory sensitivity may reflect a more stable sensory vulnerability.

Figure 1 illustrates the relationship between perceived stress and total subjective auditory sensitivity in both groups. Positive associations were observed in both the ASD group (Figure 1A) and the comparative group (Figure 1B). The correlation coefficient was numerically higher in the comparative group (rho = 0.62) than in the ASD group (rho = 0.39); however, no formal statistical comparison between these coefficients was performed.

### 3.3. Distribution of Subjective Auditory Sensitivity According to Study Groups

The distribution of total Khalfa questionnaire scores showed consistently higher values in the ASD group compared with the comparative group. This difference was statistically significant (U = 112.50, Z = −5.65, *p* < 0.001). Likewise, the ASD group showed greater dispersion in subjective auditory sensitivity scores, indicating greater within-group variability compared with participants without ASD (Figure 2).

## 4. Discussion

### 4.1. Subjective Auditory Sensitivity and Perceived Stress in ASD

The comparative group was recruited from the same university context but was not individually matched to the autistic group by sex, academic program, or mental health status. Therefore, between-group comparisons should be interpreted with the possibility of residual confounding in mind. These findings suggest that the subjective auditory sensitivity observed in participants with ASD cannot be explained solely by elevated levels of perceived stress. Although positive associations between perceived stress and subjective auditory sensitivity were observed in both study groups, participants with ASD exhibited substantially higher levels of both auditory sensitivity and perceived stress. This pattern suggests that perceived stress may be associated with auditory experience, while additional neurodevelopmental and sensory processing characteristics associated with ASD may also contribute to the elevated auditory sensitivity observed in this population. Consequently, the relationship between stress and auditory sensitivity in ASD is likely multifactorial rather than exclusively dependent on perceived stress levels.

Several studies have reported that people with ASD exhibit alterations in the modulation and processing of sensory stimuli, particularly in the auditory domain, manifested as greater reactivity to everyday sounds, difficulties with habituation, and exacerbated emotional responses to environmental acoustic stimuli [4,12,13]. These alterations have been associated with sensory overload phenomena and difficulties in auditory filtering and inhibition mechanisms, which could contribute to a greater subjective perception of auditory sensitivity compared with individuals without ASD [14,15].

Taken together, the results are consistent with evidence describing ASD as a condition characterized by greater sensory vulnerability, in which perceived stress appears to be associated with subjective auditory experience rather than as an isolated explanatory factor [16,17].

### 4.2. Possible Neurophysiological Mechanisms

One possible explanation for the observed findings could be related to alterations in auditory modulation and neurophysiological regulation mechanisms previously described in people with ASD. Several authors have proposed that the sensory alterations in this condition do not depend solely on cognitive or behavioral phenomena, but also on differences in the integration and neurophysiological control of auditory information [12,13].

From an auditory perspective, efferent and modulatory mechanisms of the auditory system have been proposed to play a significant role in filtering, inhibiting, and regulating responses to environmental acoustic stimuli. Alterations in these mechanisms could favor states of auditory hyperreactivity and a lower capacity for habituation to everyday sounds, phenomena frequently described in populations with ASD [4,18].

In this context, Sahley and Nodar proposed that certain states of physiological stress could influence auditory modulatory systems by releasing excitatory neurotransmitters and neuropeptides associated with mechanisms of auditory sensitivity, particularly glutamate and dynorphin. According to this model, the interaction between stress and peripheral auditory systems could favor states of neural hyperexcitability and greater reactivity to environmental acoustic stimuli, even without equivalent increases in the objective intensity of the sound stimulus [8].

The results of the present study are compatible with the hypothesis that the subjective experience of stress could be related to a greater perception of auditory sensitivity in students with ASD. However, because the study did not include objective neurophysiological measures, such as auditory evoked potentials, olivocochlear reflex, audiometry, otoacoustic emissions, or autonomic markers, this interpretation should be considered exploratory. Future research should directly evaluate these mechanisms to determine whether the observed association reflects processes of auditory modulation, emotional regulation, sensory hypervigilance, or other neurophysiological or psychological mechanisms.

Likewise, the significantly higher levels of perceived stress observed in participants with ASD may be associated with the elevated levels of subjective auditory sensitivity found in this group. However, the moderate magnitude of the correlations observed within the ASD group suggests that stress alone is unlikely to explain auditory hypersensitivity fully. Instead, these findings are consistent with the notion that stress-related processes may interact with neurodevelopmental and sensory processing characteristics associated with ASD, and together may be associated with the manifestation of auditory sensitivity.

It is important to consider that both groups consisted of university students exposed to similar academic demands and environmental conditions. Despite these shared contextual factors, participants with ASD reported significantly higher levels of both perceived stress and subjective auditory sensitivity. Furthermore, positive associations between stress and auditory sensitivity were observed in both groups. The students with ASD presented a markedly greater auditory vulnerability than their peers without ASD, while perceived stress appears to be an additional factor associated with a broader phenomenon likely related to neurodevelopmental and sensory processing characteristics associated with autism.

### 4.3. Clinical Implications and Future Directions

The alterations in auditory sensitivity observed in people with ASD have significant implications across different domains of everyday functioning, particularly in academic, social, and environmental contexts where there is constant exposure to complex sound stimuli. Several studies have indicated that auditory hyperreactivity and difficulties in sensory modulation may be associated with cognitive fatigue, avoidance of social environments, attentional difficulties, and increased negative emotional responses to everyday auditory stimuli [14,17].

In university populations, these difficulties may be especially relevant due to sustained exposure to acoustically demanding environments, high cognitive load, and frequent academic stress. In this context, understanding the interaction between perceived stress and subjective auditory sensitivity observed in the present study could inform the development of support strategies for sensory regulation, environmental adaptation, and the promotion of well-being in students with ASD.

Likewise, the findings of the present study could provide preliminary evidence for future research exploring objective physiological mechanisms underlying auditory sensitivity and stress in ASD. In particular, further study of efferent auditory modulation systems, especially the medial olivocochlear system, would be relevant, given its possible role in regulating, filtering, and inhibiting environmental acoustic stimuli. Similarly, future research could incorporate specific neurophysiological methodologies that allow a more precise exploration of the interaction between stress, auditory modulation, and sensory vulnerability in ASD populations.

From an applied perspective, these findings are consistent with the need for sensory-informed university accommodations, including quieter study spaces, flexible classroom arrangements, permission to use noise-reducing devices, anticipatory information about high-noise environments, and stress-management strategies tailored to autistic students.

### 4.4. Limitations

This study has several limitations. First, the sample size was small and based on non-probabilistic convenience sampling, which limits generalizability. In addition, an exploratory post hoc power analysis indicated lower statistical power for the ASD group (57.7%) reinforcing the need for cautious interpretation of the observed correlations and confirmation in larger, prospectively powered studies. Second, the cross-sectional design prevents causal or directional interpretations. Third, auditory sensitivity and perceived stress were assessed using self-report questionnaires, and no objective auditory measures, such as audiometry, otoacoustic emissions, auditory evoked potentials, or olivocochlear reflex testing, were included. Fourth, anxiety, depression, sleep quality, academic load, sensory fatigue, medication use, and severity of autistic traits were not assessed, although these factors may influence both perceived stress and auditory sensitivity. Finally, the comparative group was not matched to the autistic group by sex, academic program, mental health status, or sensory profile, and participants in the comparative group were not formally screened for autistic traits. In addition, participants with ASD were recruited through the university’s Pedagogical Support Unit, whereas the comparative group was recruited through open university-wide invitations. Although this approach facilitated identification of students with a documented ASD diagnosis, it may have introduced selection bias by preferentially including autistic students who were engaged with institutional support services. Therefore, the findings should be interpreted as preliminary, exploratory, and hypothesis-generating.

Additionally, multiple between-group comparisons and correlation analyses were conducted without formal adjustment for multiple testing. Given the exploratory nature of the study, the analyses were retained at an uncorrected significance level; however, this approach increases the possibility of false-positive findings. Therefore, findings with borderline statistical significance should be interpreted cautiously and confirmed in larger, adequately powered samples.

## 5. Conclusions

Autistic university students reported higher perceived stress and markedly greater subjective auditory sensitivity than non-autistic students. Perceived stress was positively associated with auditory sensitivity in both groups; however, a positive association was also observed in autistic students, despite being of more moderate magnitude. These findings suggest that stress-related auditory discomfort may be relevant across university populations whereas the elevated auditory sensitivity observed in autistic students may reflect a broader sensory processing vulnerability. The present findings highlight the potential value of sensory-friendly academic environments and stress-reduction strategies as part of inclusive support systems for autistic students. Future longitudinal studies incorporating objective auditory, autonomic, and psychological measures are needed to clarify the mechanisms underlying these associations.

## Figures and Tables

**Figure 1:**
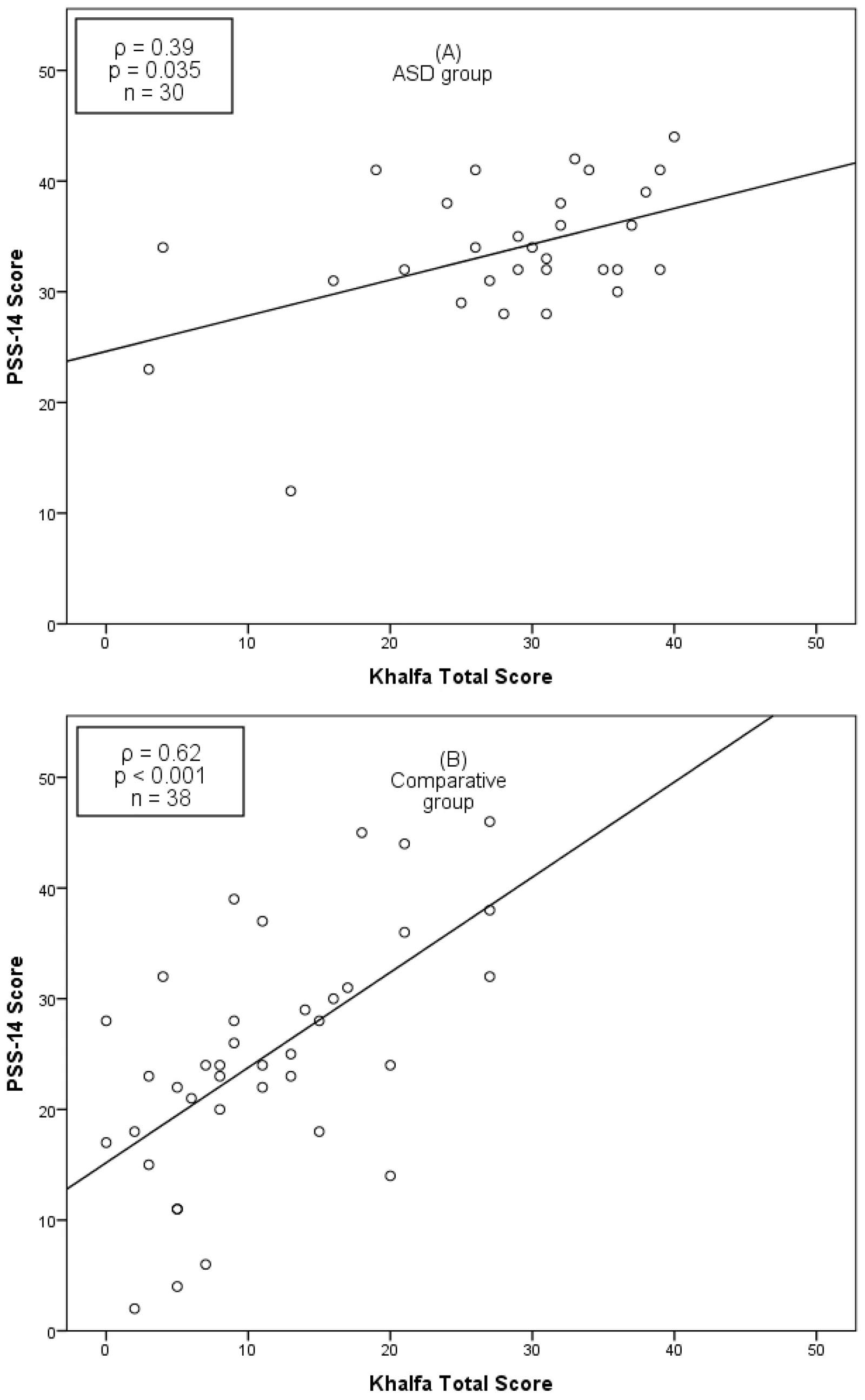
Scatterplots showing the relationship between perceived stress (PSS-14) and subjective auditory sensitivity (Khalfa Total Score) in the ASD group (**A**) and the comparative group (**B**). Positive associations were observed in both groups. The ASD group showed higher overall levels of subjective auditory sensitivity, whereas the comparative group exhibited a numerically higher correlation coefficient between perceived stress and auditory sensitivity. PSS: Perceived Stress Scale; ASD: autism spectrum disorder.

**Figure 2:**
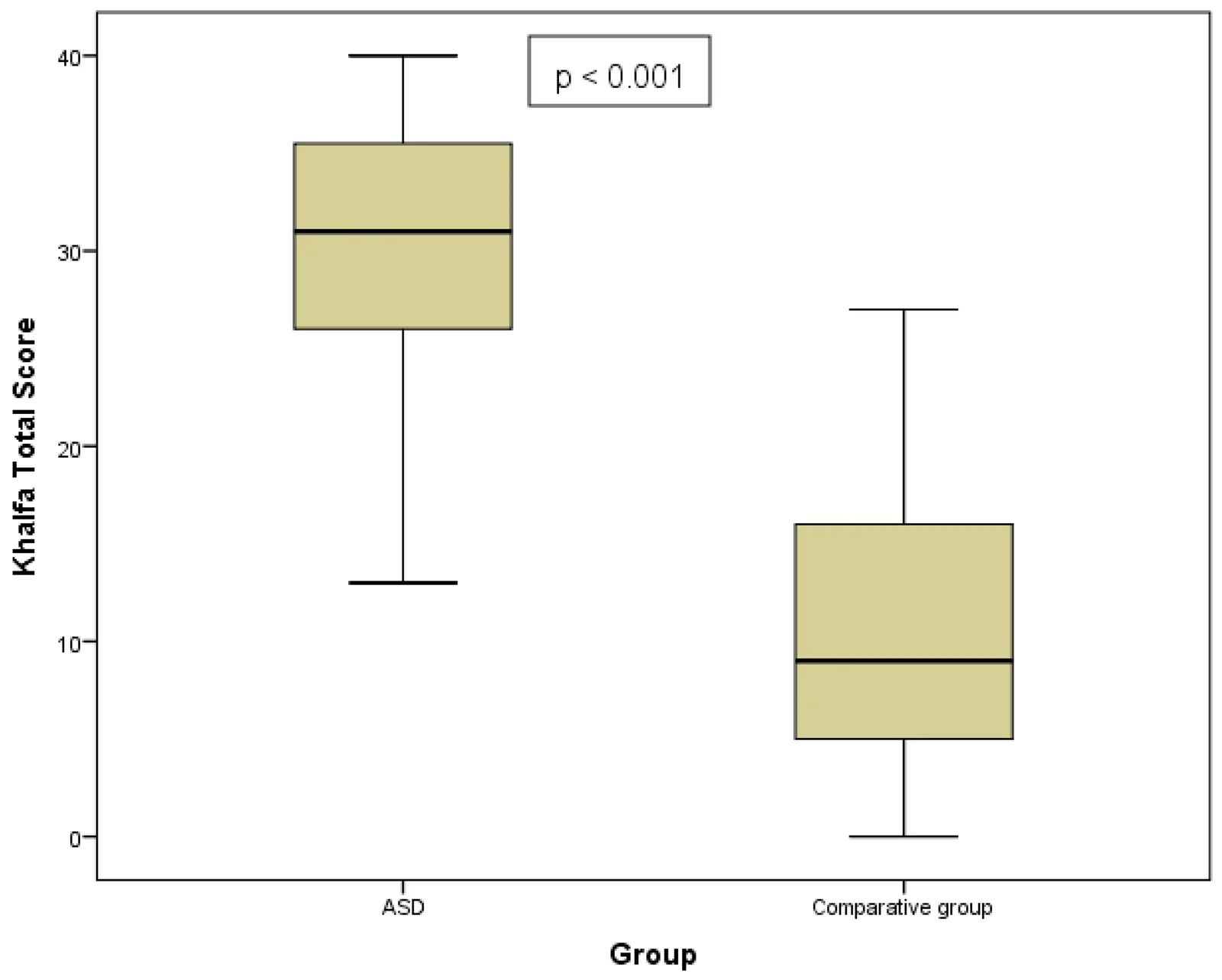
Distribution of subjective auditory sensitivity scores according to study groups. Participants with ASD showed significantly higher subjective auditory sensitivity scores than the comparative group (Mann–Whitney U = 112.50, Z = −5.65, *p* < 0.001). The ASD group showed higher total scores and greater dispersion compared with the comparative group.

**Table 1:** Sociodemographic characteristics, perceived stress, and subjective auditory sensitivity in students with ASD and the comparative group.

	ASD Group *n* = 30	Comparative Group *n* = 38	Mann–Whitney U/χ^2^	*p*-Value	Effect Size (r)
Age (mean ± SD)	22.80 ± 5.00	22.10 ± 4.90			
Age (median [IQR])	22 (19–25)	21 (20–22)			
Male sex, *n* (%)	12 (40.0%)	19 (50.0%)			
Female sex, *n* (%)	17 (56.7%)	19 (50.0%)			
Other sex, *n* (%)	1 (3.3%)				
PSS-14 (mean ± SD)	33.70 ± 6.30	24.70 ± 10.16	249.50	<0.001	0.48
Total Khalfa (mean ± SD)	28.10 ± 9.40	11.10 ± 7.50	112.50	<0.001	0.69
Attentional subcategory (mean ± SD)	7.80 ± 2.50	3.30 ± 2.10	104.00	<0.001	0.70
Social subcategory (mean ± SD)	11.10 ± 5.00	2.80 ± 3.10	114.00	<0.001	0.69
Emotional subcategory (mean ± SD)	9.10 ± 3.00	4.80 ± 3.20	186.50	<0.001	0.58

Data are presented as mean ± standard deviation, median (interquartile range), or frequency (%). PSS: Perceived Stress Scale; ASD: autism spectrum disorder. Attentional, Social, and Emotional correspond to the three dimensions of the Khalfa Auditory Sensitivity Questionnaire.

**Table 2:** Correlation between perceived stress and subjective auditory sensitivity scores in the ASD group and comparative group.

Variable	ASD Group rho	ASD Group *p*	Comparative Group rho	Comparative Group *p*
PSS vs. Attention	0.39	0.034	0.43	0.007
PSS vs. Social	0.31	0.098	0.52	0.001
PSS vs. Emotional	0.28	0.129	0.61	<0.001
PSS vs. Khalfa total	0.39	0.035	0.62	<0.001

rho: Spearman correlation coefficient; PSS: Perceived Stress Scale; ASD: autism spectrum disorder. Attentional, Social, and Emotional correspond to the three dimensions of the Khalfa Auditory Sensitivity Questionnaire.

## Data Availability

The data presented in this study are available from the corresponding author upon reasonable request, subject to the policies and confidentiality requirements established by the Scientific Ethics Committee that approved the study.
